# Supplementary material for: Population Genetic Structures of Puccinia striiformis f. sp. tritici in the Gansu-Ningxia Region and Hubei Province, China
Source: Genes (Basel). 2021 Oct 27;12(11):1712. doi: 10.3390/genes12111712 (PMC8618938; doi:10.3390/genes12111712)
Supplement: Supplementary file 1 [file genes-12-01712-s001.zip › Supplementary file 2.pdf]

Table S2. Bayesian assignments of *Puccinia striiformis* f. sp. *tritici* for 10 subpopulations collected from Hubei and the Gansu-Ningxia region of China from the 2016 fall to the 2018 spring

Subpopulation	[a]Genetic group			
	G1 (%)	G2 (%)	G3 (%)	Admixture (%)
16F-GN	10.42	40.63	32.29	16.67
16F-HB-SY	0	9.09	81.82	9.09
16F-HB-XY	0	0	86.67	13.33
16F-HB-SZ	0	0	100	0
17S-HB-SY	20.93	16.28	30.23	32.56
17S-HB-XY	7.69	0	76.92	15.38
17F-GN	17.39	30.43	30.43	21.74
18S-HB-SY	60.00	30.00	10.00	0
18S-HB-JM	0	33.33	66.67	0
18S-HB-XY	26.92	15.38	50.00	7.69

Note: [a]The percentages of isolates assigned into the G1, G2, and G3, corresponding to the blue, red and green groups in Fig 4 (C-E) with $Q > 0.8$, respectively. The admixture was the percentage of mixed isolates from G1, G2 and G3 (light gray group in Fig 4C, 4D, and 4E) with $0.2 < Q < 0.8$.
