# Supplementary figures and images for: Population Genetic Structures of Puccinia striiformis f. sp. tritici in the Gansu-Ningxia Region and Hubei Province, China
Source: Genes (Basel). 2021 Oct 27;12(11):1712. doi: 10.3390/genes12111712 (PMC8618938; doi:10.3390/genes12111712)

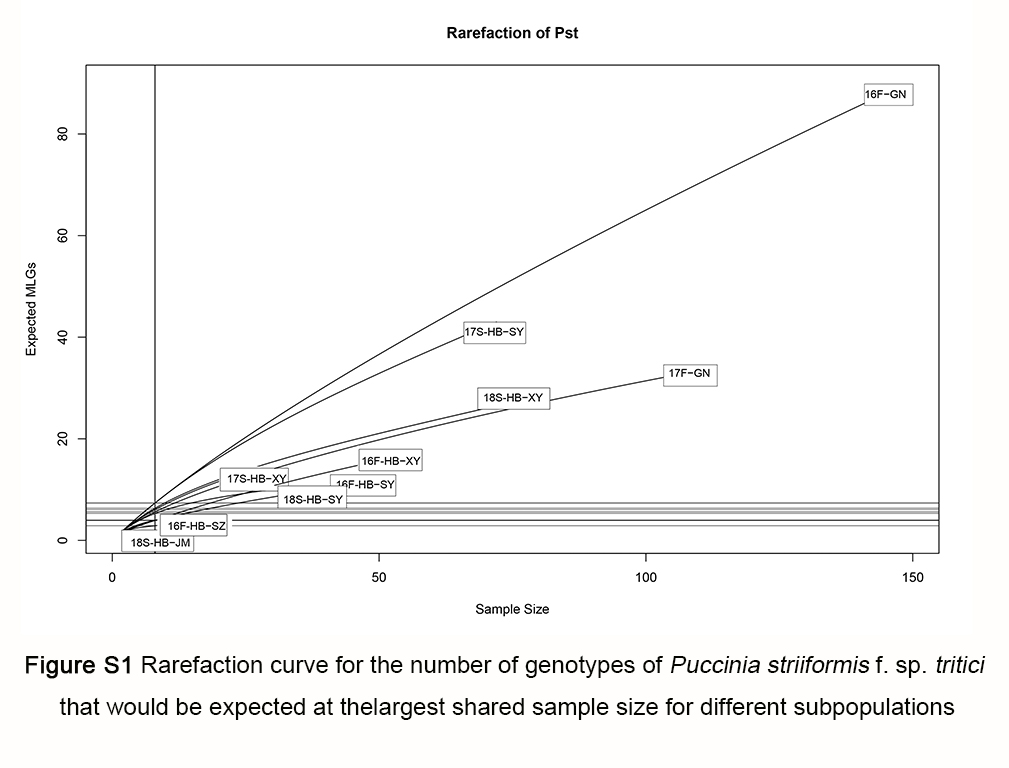

Supplement: Supplementary file 1 [file genes-12-01712-s001.zip › Figure S1.jpg]
